# Hybrid Intelligent System to Perform Fault Detection on BIS Sensor During Surgeries

**DOI:** 10.3390/s17010179

**Published:** 2017-01-18

**Authors:** José-Luis Casteleiro-Roca, José Luis Calvo-Rolle, Juan Albino Méndez Pérez, Nieves Roqueñí Gutiérrez, Francisco Javier de Cos Juez

**Affiliations:** 1 Department of Industrial Engineering, Universidade da Coruña, 15405 Coruña, Spain; 2 Departamento de Ingeniería Informática y de Sistemas, Universidad de La Laguna, Apdo. 456; 38200 La Laguna, Spain; jamendez@ull.edu.es; 3 Project Engineering Area, Department of Exploitation and Exploration of Mines, University of Oviedo, 33004 Oviedo, Spain; nievesr@uniovi.es; 4 Prospecting and Exploitation of Mines Department, University of Oviedo, 33004 Oviedo, Spain; fjcos@uniovi.es

**Keywords:** EMG, BIS, clustering, MLP, SVM, anesthesia, dosification

## Abstract

This paper presents a new fault detection system in hypnotic sensors used for general anesthesia during surgery. Drug infusion during surgery is based on information received from patient monitoring devices; accordingly, faults in sensor devices can put patient safety at risk. Our research offers a solution to cope with these undesirable scenarios. We focus on the anesthesia process using intravenous propofol as the hypnotic drug and employing a Bispectral Index (BIS^TM^) monitor to estimate the patient’s unconsciousness level. The method developed identifies BIS episodes affected by disturbances during surgery with null clinical value. Thus, the clinician—or the automatic controller—will not take those measures into account to calculate the drug dose. Our method compares the measured BIS signal with expected behavior predicted by the propofol dose provider and the electromyogram (EMG) signal. For the prediction of the BIS signal, a model based on a hybrid intelligent system architecture has been created. The model uses clustering combined with regression techniques. To validate its accuracy, a dataset taken during surgeries with general anesthesia was used. The proposed fault detection method for BIS sensor measures has also been verified using data from real cases. The obtained results prove the method’s effectiveness.

## 1. Introduction

Anesthesia control has aroused the attention of many scientists in recent years in order to move towards personalized drug infusion [1,2], where the drug dose infused to the patient is calculated according to precise individualized measures in the operating room. The three main variables that the clinician has to pay attention to during surgery are hypnosis, analgesia, and neuromuscular blockade. Hypnosis measures the patient’s unconsciousness level, analgesia is related to pain mitigation, and neuromuscular blockade refers to immobility.

Although control of analgesia is still a challenge, significant advances have been made in the automatic control of hypnosis and neuromuscular blockade [3,4]. For hypnosis control, different techniques using both signal-based and mode-based methodologies have been used successfully [5,6,7]. “Intelligent” techniques have also been implemented [8].

Most commercial infusion systems implement open-loop strategies that predict the drug concentration in the patient according to precomputed models. In these devices, there is no feedback of the real state of a patient for computing the delivered dose. A great advancement in drug infusion appears when the whole system is operated in a closed-loop. Some new devices under this methodology are starting to be available, but more advances in terms of reliability and patient-centered administration are needed.

The key point for the development of infusion systems for hypnosis control is the availability of reliable sensors to measure the patient’s state. Different techniques have been proposed to measure hypnotic depth. One method to measure the hypnotic state—based on Shannon entropy—is called spectral entropy. With this method, it is possible to predict the value of electroencephalogram (EEG) amplitude based on the distribution of the predicted variable previously detected over the signal [9,10]. It is also possible to use the Patient State Index (PSI) to quantify EEG analysis. The PSI is achieved through comparative measures of changes in brain electrical activity when the patient comes back to consciousness [11]. Auditory Evoked potentials have also been employed as a surrogate measure of the hypnotic effect [10]. More recently, a new index called QCON —obtained from the processing of the raw EEG—has been proposed to measure the patient’s hypnotic level [12].

Nonethelss, the Bispectral Index (BIS) is the most commonly accepted EEG reading of the hypnotic state. Many studies during the last 15 years have been based on the use of BIS signal to measure the influence of anesthetic drugs. The BIS is obtained through EEG processing. If the BIS signal is considered as a process value under a controlled point of view during general anesthesia, it is necessary to provide the right dose of drug to ensure that the patient’s BIS value is between 40 and 60. It is common to select 50 as the target for the controller.

The reliability of the measured BIS signal is critical to the successful implementation of sedative and anesthesia strategies. The main issue is the quality of the signal, though signal disturbances are also a concern. Like many other devices, the BIS monitor provides a signal quality index that can be used to reject poor quality signals. Different sources of disturbances can affect the signal during surgery. Most are related to the surgical procedure itself: skin incision, blood loss, interaction with other drugs, etc. The BIS can vary due to these disturbances, and the clinician has to take this into account to modify the drug dose so that the BIS reaches its target value again. Along with this, there are also disturbances that can affect raw EEG measures, and consequently the performance of BIS monitoring during surgery. The main source of these disturbances is incorrect measures of muscular relaxation in the patient. Muscular relaxation is assessed by means of the electromyogram (EMG) signal. Experience shows that inappropriate EMG episodes can affect the BIS signal. Thus, the clinician can observe a BIS value that does not correspond with the real state of the patient. Although the BIS recovers its real value after some time, it is important to detect these failures in the BIS measures so that the clinician does not make incorrect decisions based on these mistaken values, thereby avoiding overdoses or awakening episodes during surgery.

This work presents an approach based on artificial intelligence for the implementation of a fault detection system in BIS monitoring, in order to increase the reliable assessment of a patient’s hypnotic state.

Preliminary studies in this direction can be found in References [13,14], which, respectively, created a hybrid model for predicting the EMG trace using the propofol dose provided and the BIS, and predicted the BIS signal using the propofol dose and the EMG signal. In both works, a regression over a system with a very high non-linear component was necessary to model the process. Hence, clustering techniques followed by regression were applied over the resulting groups.

The approach described in the present work focuses on a robust BIS monitoring system development capable of rejecting incorrect measures due to EMG disturbances, based on the hypothesis that the BIS signal is related to EMG and the drug dose supplied.

In Reference [15], sensor fault detection is defined as a sensor malfunction that causes a deflection form in the normal reading. It can occur for several reasons: disturbances, damage to the reading device, bad sensor placement, and so on. Regardless of the solution to correct the malfunction, the first step is to detect the fault in the sensor reading because of the possible consequences on the patient. Physical redundancy could be a solution for sensor fault detection [16], but is difficult to implement because of concerns like viability, applicability, and cost. However, analytical redundancy could be applied. According to [17], it is possible to divide sensor fault detection into three main groups: classification-based fault detection, signal-based fault detection in intelligent sensors, and model-based fault detection. For the first, one usually needs to know all the possible faults. If a new fault type appears, it may not be possible to detect, because it is not part of the classification system. Signal-based fault detection usually employs correlation analysis between other sensors, which is not always possible. Because of the disadvantages of the first two methods, model-based fault detection is usually used to analytically study sensor fault detection.

There are fields where sensor fault detection was determined by employing model-based techniques. In Reference [18], current sensor fault detection and energy state estimation are performed at the same time. A new system identification technique and anomaly detection are described in Reference [19], based on the Bayesian belief network. A proposal for sensor anomaly detection in real time—based on the budding Strong Tracking Filter—is described in Reference [20]. The work in Reference [21] presents a sensor Fault Detection and Isolation implementation system for a condensation process, based on a nonlinear model.

The anesthetic state is a very delicate process, because the patient’s life could be at risk if the professional responsibility for this task does not proceed suitably. As was explained previously, the present work focuses on the anesthesia process when the patient is monitored through BIS signal. It is mandatory to ensure correct sedation, and obviously, doing so is linked to correct sensor operation. Whether the anesthesia process is manual or automatic, the detection of BIS sensor malfunctions is essential. The aim of the present research is to implement a fault detection system for BIS sensors during surgery; in order to accomplish this goal, a hybrid intelligent system based on sensor fault detection was developed.

Many methods can be used to model sensor fault detection. Multiple regression analysis (MRA) techniques are the base of most accepted regression methods [22,23,24,25]. Despite the limitations and poor performance of these techniques, they are widely used in many different applications [26]. Soft computing techniques are used with the aim of increasing performance. Simple or hybrid proposals are used in order to improve MRA techniques [27,28,29,30].

Our BIS sensor fault detection system is based on the propofol drug infusion rate and the EMG signal. In developing the model, several regression techniques were applied to the data, which is divided into groups. The data groups were created using a K-means clustering algorithm. This algorithm divides the data into groups with similar behavior. Once the regression methods were applied to each group, the results were compared by the mean squared error (MSE), and we chose the data set providing the lowest MSE.

This paper is divided as follows: after this introduction, the Bispectral Index is introduced. Then, the model approach and the tested algorithms taken into account by our research are shown. The obtained results are described, identifying the best configuration achieved by the hybrid model. Finally, conclusions are drawn, and future works are presented.

## 2. The Anesthesia Issue

The sensor fault detection system proposed in this work was designed to be used in the operating room when a patient is under surgery. Three main variables related to the anesthetic process are under medical control: hypnosis, analgesia, and muscular relaxation. The drugs used to regulate each variable are propofol, remifentanil, and rocuronium, respectively. All are administered intravenously. This paper focuses on hypnosis monitoring using the BIS signal. The objective is to use intelligent techniques to predict the patient’s BIS signal using information on the EMG and the propofol infusion rate. As is well known, remifentanil also influences the BIS signal. In this work, we will assume that the remifentanil infusion rate is kept constant and at a low dose throughout the process. That is why remifentanil is not explicitly considered as an input to the system. To achieve the proposed objective, a fault detection algorithm will be implemented to reject incorrect BIS measures.

### 2.1. Sensor Technology for Hypnosis Assessment

**Figure 1:**
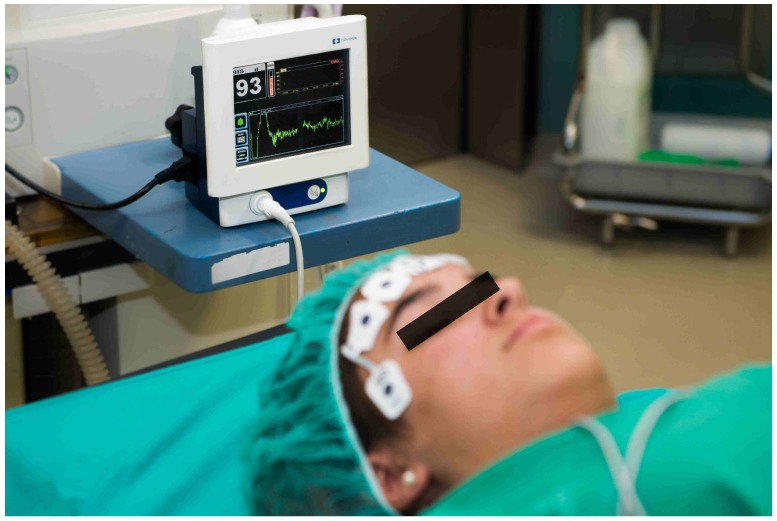
Bispectral Index (BIS) Vista monitor and a volunteer.

As commented, one of the most extended monitoring systems for the measurement of the level of unconsciousness in a patient is the BIS monitor. The BIS is obtained from the EEG signal, and consists of a sensor, a digital signal converter, and a monitor. The sensor is placed in the forehead of the patient (see Figure 1) to register the cerebral cortex electric activity. A combination of bispectral analysis, power spectral analysis, and time domain analysis is applied to the captured raw EEG signal. Then, an algorithm that obtains the optimum combination of these features using multivariate statistical modeling techniques is applied to obtain the BIS signal. As a result, an adimensional index whose value fluctuates between 100 (awake state) and 0 (maximal effect, brain without electrical activity) is obtained [31]. A BIS range from 60 to 80 is considered a state of sedation. When the BIS signal is between 40 and 60, it corresponds to general anesthesia region. The monitor includes a preprocessing subsystem to detect artifacts. Potential artifacts may be caused by poor skin contact (due to high impedance), muscle activity or rigidity (due to inadequate muscular blockade), head and body motion, sustained eye movements, improper sensor placement, and excessive electrical interference. Some of these artifacts can be detected by the monitor, but some others cannot be efficiently detected, so the resulting BIS signal should be interpreted with caution.

Currently, four different types of sensors are available for different clinical uses:

- BIS^TM^ Quatro Sensor: four-electrode sensor for general use.
- BIS^TM^ Bilateral Sensor: for advanced monitoring applications. It can detect hemispheric differences in the brain.
- BIS^TM^ Pediatric Sensor: for pediatric patients.
- BIS^TM^ Extended Sensor: for use in ICU where long monitoring periods are common.

In this work, a BIS Vista monitor using a Quatro Sensor with four ZipPrep electrodes (both from Aspect Medical System, Newton, MA, USA) was used to measure the BIS signal. The BIS monitor provides the BIS signal using a RS232 port connection to a laptop.

On the other hand, the laptop communicates with the propofol infusion pump to monitor the infused drug dose. The pump used was a Graseby 3500 infusion pump (Graseby Medical Ltd., Watford, UK) administering propofol 1%, also connected via RS232 port to the laptop. Monitoring software processed the information received from the devices and implemented the fault detection system. When a fault was detected, a warning was activated, and appropriate actions were taken.

### 2.2. Clinical Study and Protocol

This study considered a population of ASA I-II patients undergoing general anesthesia. The ASA score is the American Society of Anesthesiologists’ physical status classification system—a scoring system that has some correlation with potential patient surgery risks according to their medical status or similar. ASA I corresponds to a normal healthy patient, and ASA II to a patient with mild systemic disease. Patient population age was in the range 18–80 years. The criteria for exclusion were refusal by the patient, ASA ≥ III, psychiatric or neurological disorder, or the presence of a pacemaker.

**Figure 2:**
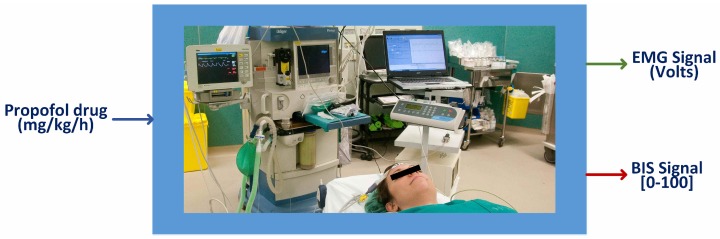
The anesthesia issue with a volunteer. Input and Output definition.

For the clinical setup, an intravenous cannula was placed with an antireflux valve in the preoperative room, and a single midazolam dose of 0.02 mg/kg was provided. The remifentanil infusion was started at 0.25 mcg/kg/min at least 5 min before intubation. The patients received for induction a propofol bolus of 2 mg/kg and rocuronium bromide (0.6 mg/kg). Once the patients lost consciousness and their BIS value was around 50, they were intubated, and mechanical ventilation was maintained with oxygen + air (40%). Ten minutes after the initial dose, the infusion of remifentanil was decreased to 0.15 mcg/kg/min. This value was kept constant unless the patient’s analgesic state required a variation. Approximately 15–20 min before the end of the surgery, rescue analgesia was administered with fentanyl (1.5 mcg/kg) and dexketoprofen (50 mg·iv) or paracetamol (1 gr·iv). To finish the surgery, propofol was stopped. The input–ouput representation of the patient in the operating room is shown in Figure 2.

## 3. Hybrid Model Proposal

**Figure 3:**
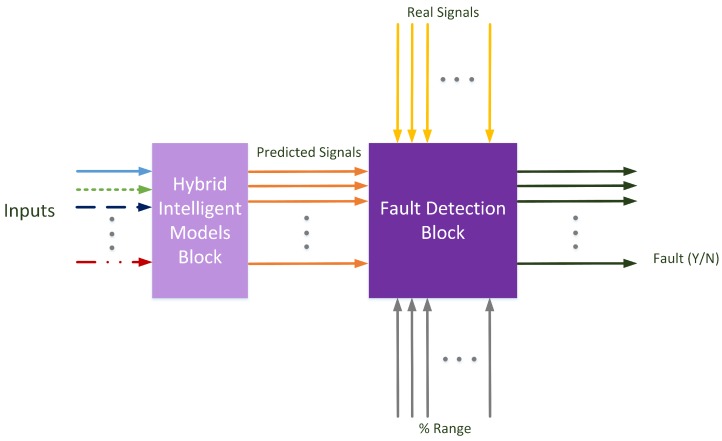
Hybrid model proposal.

In this article, the modeled approach is defined as shown in Figure 3. In this figure, two main blocks have been created: a hybrid intelligent models block and a fault detection block. The first one includes the models for the different signals to control. The models used in this research are hybrid intelligent models, and for each predicted signal, a complete model was trained. Each model uses the necessary inputs to calculate the specific predicted signal.

The second block in Figure 3 is the one where the fault detection algorithm is implemented. The signals predicted by the hybrid intelligent models are compared with the real measured signals. Generally speaking, each signal has a specific range for detecting—or failing to detect—a fault in the signal. This range is demarcated as the allowed signal variation before a fault situation is set. In other words, it is the predicted error allowed by the system.

**Figure 4:**
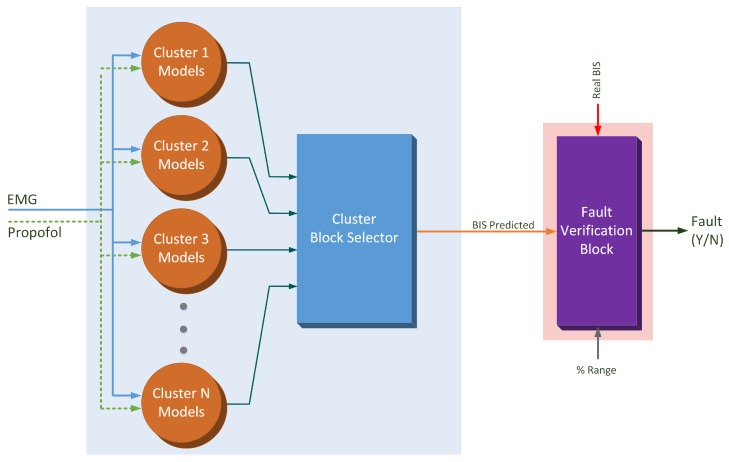
BIS case. EMG: electromyogram.

In Figure 4, the model used to predict the BIS signal is shown. The hybrid intelligent model consists of a set of local models valid for different situations of operation. As explained above, two input signals are fed into the models block: the EMG measured signal and the propofol infusion rate. The predicted BIS output is obtained as the output of the local model associated with the current situation. Once the predicted BIS signal is calculated, this signal is compared with the real BIS signal. Considering the specific range of possible error, an eventual fault situation can be detected.

**Figure 5:**
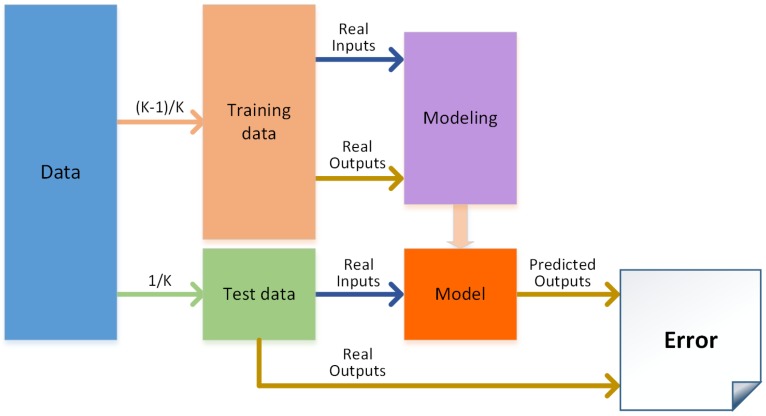
Modeling process.

Figure 5 shows the modeling process used to create each local intelligent model. To ensure better performance, the models were created using k-fold cross validation for each of the algorithms tested. Once all the folds were tested, the performances for all the algorithms were compared, and only the best configuration was selected.

Once the error value was computed for each cluster using the MSE is as a performance measurement, each local model’s combination error was calculated. This calculation took into account the number of samples in each cluster to ensure a valid performance comparison. The above modeling procedure was applied for each cluster, and for the best local model combination. After the selection of the best model, all the data in the cluster was used to train the best algorithm selected.

A fault situation means that an unexpected value is measured in the BIS sensor. Different reasons can lead to this situation. The most common could be a malfunction in this sensor. Another possibility is that the measured BIS value is correct, but affected by other disturbances, such as an improper neuromuscular blockade. For instance, a sudden rise in the BIS signal can occur due to an increase in the EMG if a neuromuscular blockade is not correctly implemented. This increase is not related to the real hypnotic state of the patient, so the clinician should reject the measured BIS values when defining the propofol dose. A third possibility is that the the fault could be caused by an unusual patient reaction during the surgery, indicating a possible ‘patient fault’ and not a ‘sensor fault’. Thus, the system could be used not only to perform fault detection on the sensors used in surgeries, but to detect an abnormal patient reaction. In this situation, the clinicians are warned to check the patient’s state to ensure that the patient is not suffering any complication during the surgery.

It is important to note that the models were created with a dataset of healthy patients (ASA I/II following the ASA physical status classification system). If, for a specific patient, the system continuously detects faults in the evolution of the BIS signal, this information may be used to detect the presence of an undiagnosed pathology. One limitation of the proposed approach is related to interpatient variability. Thus, even if the models offer very good prediction performance, a specific patient could react in different ways to the same stimulus. In such cases, the system will indicate a potential fault. The clinician must analyze the situation to make the correct decision.

### 3.1. The Dataset

In order to obtain the data used in this research, three variables (BIS, EMG, and the propofol infusion rate) were monitored in several patients during surgery. Propofol was used as the surgical anesthesia. The variables for BIS and EMG were preconditioned. In total, 50 patients were monitored to obtain the data. Due to the slow variations of the signals at the acquisition phase, measurement noise is superposed to the signals; thus, a low-pass filter was implemented for rejecting data. This study is focused on the results during the maintenance phase, since the induction and recovery phases were not considered. Consequently, the prediction of the EMG is valid only for the maintenance phase. The total dataset was built from 42,788 samples gathered according to the conditions detailed above.

### 3.2. Modelling Techniques

#### 3.2.1. Data Clustering: The K-Means Algorithm

Clustering is a technique used for grouping data. These groups contain data samples with similarities between them [32,33]. Clustering algorithms are unsupervised algorithms that organize unlabeled feature vectors into clusters or groups [33]. One of these clustering algorithms is K-means. It uses the square error to minimize the error function, as shown in Equation (1). This algorithm is a partitional clustering algorithm.

(1) $$e=\sum_{k=1}^{C}\sum_{x\epsilon Q_{k}}{∥x-c_{k}∥}^{2}$$

where *x* is every sample, $c_{k}$ is the closest cluster centroid for each case, $Q_{k}$ is the number of samples in each cluster, and *C* is the number of clusters.

The number of clusters, K, and the initial cluster centroids determine the final clustering. Determining the number of clusters requires prior knowledge of the data. This task is the most critical, because the data is highly doubtful. With the aim of obtaining an effective clustering, two conditions must be fulfilled: the data has to be close enough to its cluster, and the cluster must be hyperspherical in shape and well-separated in the hyperspace.

#### 3.2.2. Multi Layer Perceptron (MLP)

Based on a feedforward Artificial Neural Network (ANN), a Multi Layer Perceptron (MLP) [34,35,36] was defined. Thanks to its sturdiness and comparatively simple configuration, this kind of ANN is typical. Nevertheless, the architecture of the ANN must be well chosen to achieve satisfactory results. The composition of this type of ANN has an input layer, an output layer, and one or more hidden layers. The layers are composed of neurons that have activation functions. This activation function can be tan-sigmoid, log-sigmoid, linear, or step. Usually, all the activation functions of a layer are the same. The tan-sigmoid function is shown in Equation (2).

(2) $$F\left(t\right)=\frac{e^{t}-e^{-t}}{e^{t}+e^{-t}}$$

The neurons between layers are linked through weighted connections.

It is possible to define the output of an MLP as shown in Equation (3) [37].

(3) $$f_{\theta }\left(x\right)=\beta +\sum_{i=1}^{k}a_{i}\phi \left(w_{i}^{T}x+b_{i}\right)$$

where $x={\left(x\left(1\right),...,x\left(d\right)\right)}^{T}\in {ℜ}^{d}$ is the inputs vector, *k* is the hidden layers number, *ϕ* is a bounded transfer function, $\theta =(\beta ,a_{1},...,a_{k},b_{1},...,b_{k},w_{11},...,w_{kd})$ is the model parameter vector, *β* is the “offset” for all the net, $a_{i}$ are the parameters that relate each layer with the next one, $b_{i}$ is the “offset” for each layer, and $w_{i}={\left(w_{i1},...,w_{id}\right)}^{T}\in {ℜ}^{d}$ is the parameter vector for the hidden unit *i*.

#### 3.2.3. Support Vector Regression (SVR), Least Square Support Vector Regression (LS-SVR)

The Support Vector Regression is based on a classification algorithm—the Support Vector Machine (SVM)—which was modified for regression purposes. The SVM uses non-linear mapping of the data into a high-dimensional feature space, *F*, then applies linear regression in this space [38,39,40,41].

A further modification of this method is the least square formulation of SVM (LS-SVM). In this case, a system of linear equations is solved with the aim of obtaining an approximation of the solution. This modification provides generalization performance comparable to that of the SVM [42,43]. LS-SVR is the given name of the application of LS-SVM to regression [44,45]. A classical squared loss function is used instead of the insensitive loss function when the LS-SVR is applied. With this change, a linear KarushKuhn–Tucker is solved in order to build the Lagragian:

(4) $$\left[\begin{array}{cc} 0 & I_{n}^{T}\\ I_{n} & K+\gamma_{-1}I \end{array}\right]\left[\begin{array}{c} b_{0}\\ b \end{array}\right]=\left[\begin{array}{c} 0\\ y \end{array}\right]$$

where $I_{n}$ is a vector of *n* ones, ${}^{T}$ means transpose of a matrix or vector, *γ* is a weight vector, *b* is the regression vector, $b_{0}$ is the model offset, and *y* is the desired output. The weight vector (*γ*) as well as the width of the kernel (*σ*) are the only variables needed in LS-SVR [45].

#### 3.2.4. Polynomial Regression

The polynomial regression model could be defined as the summation of basis functions [46,47,48,49,50]. The quantity of inputs determines the number of basis functions needed and the polynomial degree.

Equation (5) shows a degree one case defining the linear summation for two inputs, $x_{1}$ and $x_{2}$. If the degree of the model increases, it becomes more complex; for instance, Equation (6) is the equation for degree two, where it is possible to verify the increased complexity.

(5) $$F\left(x\right)=a_{0}+a_{1}x_{1}+a_{2}x_{2}$$

(6) $$F\left(x\right)=a_{0}+a_{1}x_{1}+a_{2}x_{2}+a_{3}x_{1}x_{2}+a_{4}x_{1}^{2}+a_{5}x_{2}^{2}$$

## 4. Results

The dataset used to create the models was processed to include the two previous values of all the signals—the EMG, the propofol dosage rate, and the BIS—in each sample. This process considers the dynamics of the anesthetic process to create a more accurate model. Then, each sample for the model must include eight inputs and one output: the current and two previous values of the EMG, the current and previous values of the propofol dosage rate, and the two previous values of the BIS signal.

The output of the model is the current value of the BIS signal. Different simulation experiments were conducted to confirm that these values are sufficient to train the dynamics of the system. Our results demonstrated that the inclusion of larger regression vectors for the inputs is not necessary to improve the model accuracy.

### 4.1. Clustering and Modeling Results

The first step is to perform the clustering. During this process, the initial state must be taken into account, as the performance of the K-means algorithm is highly influenced by this condition. Random initialization was used to perform the process, which was carried out 20 times. The clusters that minimized the error function were stored. Different configurations were tested, from two to ten clusters. These configurations were tested because the number of clusters is unknown at the beginning, so the model was trained for all cases. In total, ten different topologies were created, including nine different topologies from the clusters and the global model.

Remarkably, for some clustering configurations, it was impossible to find a suitable dataset division. This limitation was caused by the constraint based on the minimum number of samples that each cluster should have: the procedure to create the clusters detects when a cluster is smaller than 15 samples. When the k-fold cross-validation is used, no cluster with less than 15 samples is considered valid. Table 1 shows the number of samples in each cluster. In each cluster, the models were trained using five k-fold cross-validations to calculate the MSE to perform the statistical analysis.

The MLP-ANN regression algorithm consists of one hidden layer at which the number of neurons used for the regression changes from 1 to 10, providing several algorithm configurations. Furthermore, these neurons are activated as a tan-sigmoid function, even though the output layer is activated by a linear function. To perform the training, the Levenberg–Marquardt algorithm was used. Moreover, the learning algorithm was the gradient descent, and with the aim of measuring the performance, the mean squared error method was fixed.

A self auto-tuning developed by KULeuven-ESAT- —which is implemented in the MatLab toolbox—was used to train the LS-SVR. The settings used to perform this training were the radial basis function (RBF) for the kernel of the model and “function estimation” to perform regression. Finally, the optimization function was set to “simplex”, while “leaveoneoutlssvm” with “mse” was used as cost criterion.

The next method used to create the model was polynomial regression. The polynomial was trained for first- to second-order regressions.

Table 2 shows the results obtained for each regression technique applied to each cluster and testing every configuration. The performance is measured by the MSE. The average value of the MSE obtained is shown in the column on the right. This means that MSE is calculated considering the number of samples in each cluster to ensure a real measure of the MSE for each configuration. As mentioned, the model is not trained in all the clusters, because the combinations shown include a cluster with fewer samples than the minimum set (represented as N/A in the table).

After testing all regression techniques for all clusters, Table 3 summarizes the best model and its configuration. Table 2 and Table 3 show that the division of the data into two clusters provides the best performance for the model. Table 4 presents a summary of the modelling results.

Once the best model was selected, the final hybrid intelligent model was created, using all the data to train the model with the corresponding algorithm.

### 4.2. Fault Detection System Results

**Figure 6:**
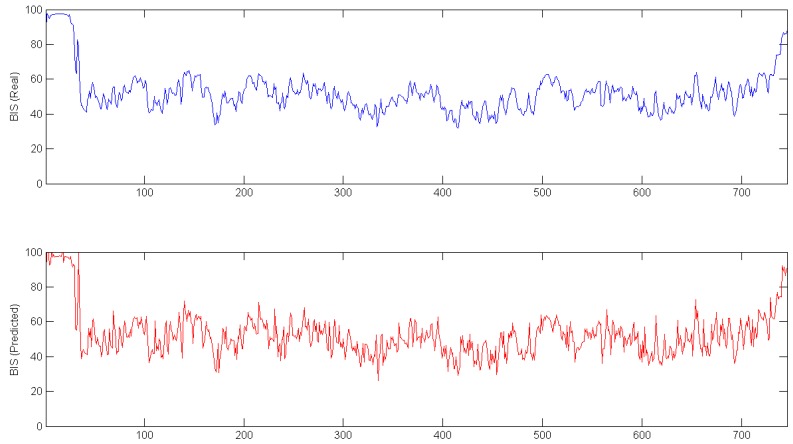
Real (**Top**); and predicted (**Bottom**) BIS signal for one complete surgery.

To ensure the accuracy of the fault detection system, the data from one complete surgery was isolated from the modeling procedure. This data was used as input to calculate a predicted BIS signal for the whole surgery. In Figure 6, the real signal (top plot) and the predicted signal (bottom plot) are shown.

**Figure 7:**
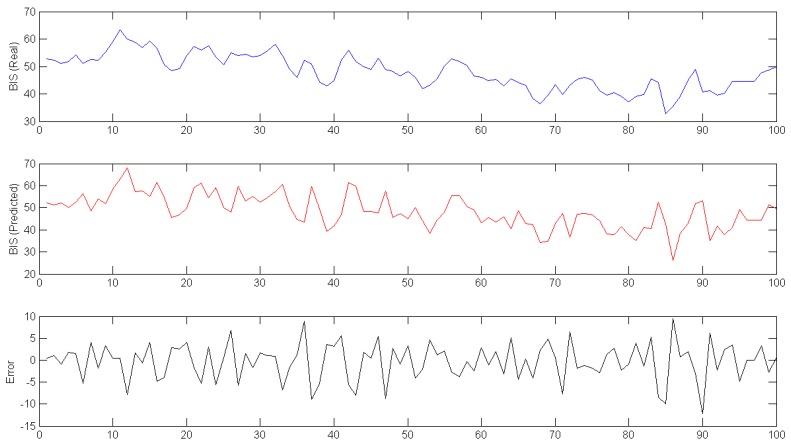
Real (**Top**); Predicted (**Middle**); and Error (**Bottom**) BIS signal for 100 samples during surgery.

In Figure 6, data from all of the surgeries is shown; however, to increase the accuracy of the model, the initial and final parts of the data were eliminated from the dataset. Figure 7 shows the difference between real and predicted data in the middle of the surgery; the third plot represents the error between the other two signals.

The limits used in Figure 7 are selected as the percentage range for the model shown in Figure 4, but this value could change during surgery. For the BIS signal, the range is shown in absolute values, since that signal has no units and varies between 0 and 100. In this case, the range of failure detection is absolute (a percentage of the total valid limits for this signal).

**Figure 8:**
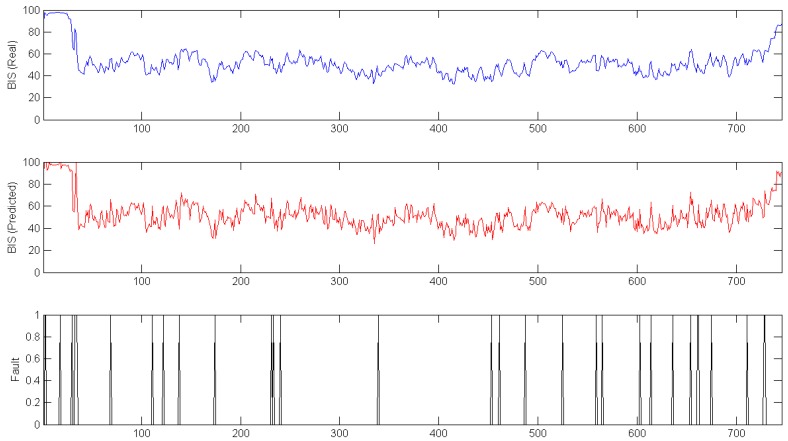
Real (**Top**); Predicted (**Middle**); and Fault (**Bottom**) Complete surgery with fault detection Range of 10.

**Figure 9:**
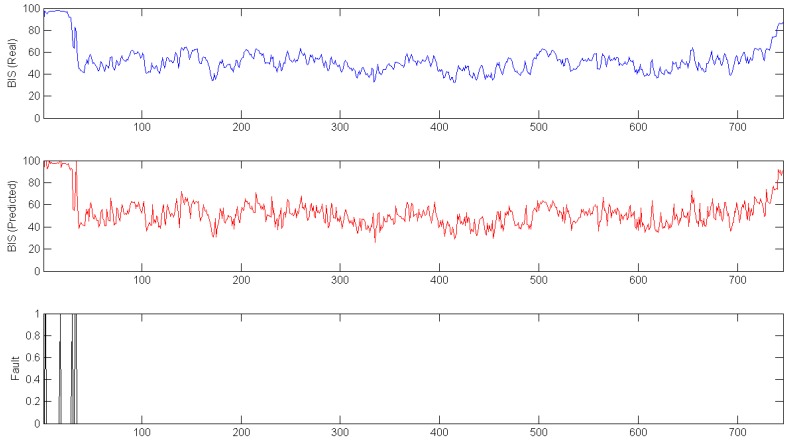
Real (**Top**); Predicted (**Middle**); and Fault (**Bottom**) Complete surgery with fault detection Range of 15.

In Figure 8 and Figure 9, the previously shown data is used to test the fault detection model. The top plots represent the real BIS signals, the middle plots show the BIS signals predicted by the hybrid intelligent model, and the bottom plots are the fault detection system. For Figure 8, the range used was only 10, and some failures appeared during surgery. However, when this range increased to 15 (Figure 9), the failures appeared only at the first stage of the surgery. This is a known limitation, as the data to train the model do not include this part of surgery.

## 5. Conclusions

This study provides a methodology for the detection of failure episodes in BIS sensors affected by disturbances. The proposal is based on a hybrid intelligent model to predict the BIS based on the EMG signal and propofol dosage provided to the patient. Once the BIS signal is predicted, it is compared with the real BIS signal measured by the sensor. The results of this comparison indicate whether a fault state may be activated.

The modelling part of the approach is based on a hybrid intelligent system, combining different regression techniques on local models.

This model was obtained from a real dataset. After testing, the analysis of the results shows that the best model configuration has two clusters. The regression techniques employed on the clusters were ANN with different configurations (two and nine neurons in the hidden layer). The best mean MSE obtained with this configuration was 0.4097. The k-fold cross validation procedure ensures a more realistic MSE, because all of the data was used for training and for testing at the end of the modelling phase.

The fault detection system can select the range for detecting failures depending on the situation. Moreover, the system could change to detect a failure not only when one measurement is out of the expected range, but when there are two or three consecutive measurements out of range. The validation of the algorithm with real data offered satisfactory results for the detection of faults in the BIS signal during surgery. The importance of the proposed methodology is that the clinician (or even an automatic infusion system) can have information of anomalous values of the BIS signal during surgery. This will improve the safety of patients in the operating room, as the drug infusion depends on the information extracted from the monitoring system.

The proposed method opens new future research lines. On one hand, other scenarios can be considered: for instance, a varying remifentanil infusion. This will require the implementation of a new model using the remifentanil infusion as an input. Additionally, extension to inhalatory anesthesia can be explored. From a more general perspective, the proposed method can be extended to other sensor devices for health monitoring so that quality of patient monitoring will increased.

## Figures and Tables

**Table 1:** Number of samples in each cluster in percentage.

**N**${}^{\circ }$ **of Clusters**	**Cluster 1**	**Cluster 2**	**Cluster 3**	**Cluster 4**	**Cluster 5**
**Global Model**	100%	-	-	-	-
2	0.8%	99.2%	-	-	-
3	0.4%	0.7%	98.9%	-	-
4	0.4%	0.7%	38.3%	60.6%	-
5	0.4%	0.7%	1.7%	40.6%	56.7%
6	0.2%	0.4%	0.7%	1.7%	41.0%
7	0.2%	0.3%	0.3%	0.7%	1.7%
8	0.1%	0.2%	0.2%	0.3%	0.7%
9	0.1%	0.2%	0.2%	0.3%	0.7%
10	0.1%	0.2%	0.2%	0.3%	0.7%
**N**${}^{\circ }$ **of Clusters**	**Cluster 6**	**Cluster 7**	**Cluster 8**	**Cluster 9**	**Cluster 10**
**Global Model**	-	-	-	-	-
2	-	-	-	-	-
3	-	-	-	-	-
4	-	-	-	-	-
5	-	-	-	-	-
6	56.0%	-	-	-	-
7	41.1%	55.7%	-	-	-
8	1.7%	41.1%	55.7%	-	-
9	1.7%	27.2%	31.3%	38.4%	-
10	1.7%	13.1%	19.2%	24.8%	39.7%

**Table 2:** Clusters mean squared error (MSE).

**N**${}^{\circ }$ **of Clusters**	**Cluster 1**	**Cluster 2**	**Cluster 3**	**Cluster 4**	**Cluster 5**	**Clusters MSE**
**Global Model**	0.8531	-	-	-	-	0.8531
2	$8.15\times 10^{-4}$	0.4129	-	-	-	0.4097
3	N/A	N/A	N/A	-	-	N/A
4	N/A	N/A	N/A	N/A	-	N/A
5	N/A	N/A	N/A	N/A	N/A	N/A
6	N/A	N/A	N/A	N/A	N/A	N/A
7	N/A	N/A	N/A	N/A	N/A	N/A
8	N/A	N/A	N/A	N/A	N/A	N/A
9	N/A	N/A	N/A	N/A	N/A	N/A
10	N/A	N/A	N/A	N/A	N/A	N/A
**N**${}^{\circ }$ **of Clusters**	**Cluster 6**	**Cluster 7**	**Cluster 8**	**Cluster 9**	**Cluster 10**	**Clusters MSE**
**Global Model**	-	-	-	-	-	0.8531
2	-	-	-	-	-	0.4097
3	-	-	-	-	-	N/A
4	-	-	-	-	-	N/A
5	-	-	-	-	-	N/A
6	N/A	-	-	-	-	N/A
7	N/A	N/A	-	-	-	N/A
8	N/A	N/A	N/A	-	-	N/A
9	N/A	N/A	N/A	N/A	-	N/A
10	N/A	N/A	N/A	N/A	N/A	N/A

**Table 3:** Best regression technique for each cluster. ANN: Artificial Neural Network.

**N**${}^{\circ }$ **of Clusters**	**Cluster 1**	**Cluster 2**	**Cluster 3**	**Cluster 4**	**Cluster 5**
**Global Model**	**ANN-06**	-	-	-	-
2	**ANN-02**	**ANN-09**	-	-	-
3	N/A	N/A	N/A	-	-
4	N/A	N/A	N/A	NA	-
5	N/A	N/A	N/A	NA	N/A
6	N/A	N/A	N/A	NA	N/A
7	N/A	N/A	N/A	NA	N/A
8	N/A	N/A	N/A	NA	N/A
9	N/A	N/A	N/A	NA	N/A
10	N/A	N/A	N/A	NA	N/A
**N**${}^{\circ }$ **of Clusters**	**Cluster 6**	**Cluster 7**	**Cluster 8**	**Cluster 9**	**Cluster 10**
**Global Model**	-	-	-	-	-
2	-	-	-	-	-
3	-	-	-	-	-
4	-	-	-	-	-
5	-	-	-	-	-
6	N/A	-	-	-	-
7	N/A	N/A	-	-	-
8	N/A	N/A	N/A	-	-
9	N/A	N/A	N/A	NA	-
10	N/A	N/A	N/A	NA	N/A

**Table 4:** Results of the chosen configuration. LS-SVR: Least Square Support Vector Regression.

	Big-Cluster	Small-Cluster
ANN	9 neurons	2 neurons
Polynomial	1st order	1st order
ANN (MSE)	0.4129	$8.15\times 10^{-4}$
LS-SVR (MSE)	0.8855	0.1680
Poly (MSE)	$1.45\times 10^{11}$	$3.34\times 10^{3}$
